# Intratumor heterogeneity comparison among different subtypes of non-small-cell lung cancer through multi-region tissue and matched ctDNA sequencing

**DOI:** 10.1186/s12943-019-0939-9

**Published:** 2019-01-09

**Authors:** Yaxiong Zhang, Lianpeng Chang, Yunpeng Yang, Wenfeng Fang, Yanfang Guan, Aiwei Wu, Shaodong Hong, Huaqiang Zhou, Gang Chen, Xi Chen, Shen Zhao, Qiufan Zheng, Hui Pan, Lanjun Zhang, Hao Long, Haoxian Yang, Xin Wang, Zhesheng Wen, Junye Wang, Hong Yang, Xuefeng Xia, Yuanyuan Zhao, Xue Hou, Yuxiang Ma, Ting Zhou, Zhonghan Zhang, Jianhua Zhan, Yan Huang, Hongyun Zhao, Ningning Zhou, Xin Yi, Li Zhang

**Affiliations:** 1 Department of Medical Oncology, Sun Yat-sen University Cancer Center, State Key Laboratory of Oncology in South China, Collaborative Innovation Center for Cancer Medicine, 651 Dongfeng Road East, Guangzhou, Guangdong 510060 People’s Republic of China; 2 Geneplus-Beijing Institute, Beijing, China; 3 Department of Thoracic Surgery, Sun Yat-sen University Cancer Center, State Key Laboratory of Oncology in South China, Collaborative Innovation Center for Cancer Medicine, Guangzhou, China; 4 Department of Clinical Research, Sun Yat-sen University Cancer Center, State Key Laboratory of Oncology in South China, Collaborative Innovation Center for Cancer Medicine, Guangzhou, China

**Keywords:** NSCLC, Multi-region sequencing, Intratumor heterogeneity, ITH, ctDNA

## Abstract

Understanding of intratumor heterogeneity (ITH) among different non-small cell lung cancer (NSCLC) subtypes is necessary. Whether circulating tumor DNA (ctDNA) profile could represent these ITH is still an open question. We performed 181 multi-region tumor tissues sequencing and matched ctDNA sequencing from 32 operative NSCLC to compare ITH among different NSCLC subtypes, including EGFR-mutant lung adenocarcinoma (LUAD), KRAS-mutant LUAD, EGFR&KRAS-wild-type LUAD, and lung squamous cell carcinoma (LUSC), and examine potential value of ctDNA for ITH analysis. ITH is evaluated by ITH index (ITHi). If the somatic genetic alteration is shared by all the tissue regions, it is defined as trunk mutation. Otherwise, it is called branch mutation. The ITHi will be higher, if the tumor has less trunk mutations. We found EGFR-mutant LUAD showed significantly higher ITHi than KRAS-mutant LUAD/wild-type LUAD (*P* = 0.03) and numerically higher ITH than LUSC. For trunk mutations, driver mutations were identified at a higher proportion than passenger mutations (60% vs. 40%, *P* = 0.0023) in overall, especially in EGFR-mutant LUAD (86% vs. 14%, *P* = 0.0004), while it was opposite in KRAS-mutant LUAD (40% vs. 60%, *P* = 0.18). For branch mutations, the proportions of driver mutations and passenger mutations were similar for each NSCLC subtype. ctDNA analysis showed unsatisfactory detections of tumor-derived trunk and branch mutations (43% vs. 23%, *P* = 4.53e-6) among all NSCLC subtypes. In summary, EGFR-mutant LUAD has the highest ITH than other NSCLC subtypes, offering further understanding of tumorigenesis mechanisms among different NSCLC subtypes. Besides, ctDNA maybe not an appropriate method to reflect ITH.

**Electronic supplementary material:**

The online version of this article (10.1186/s12943-019-0939-9) contains supplementary material, which is available to authorized users.

## Main text

Lung cancer is the leading cause of cancer-related mortality worldwide. [1] Non-small cell lung cancer (NSCLC) comprises the majority of pathological types, including epidermal growth factor receptor (EGFR)-mutant lung adenocarcinoma (LUAD), kirsten rat sarcoma viral oncogene (KRAS)-mutant LUAD, EGFR&KRAS-wild-type LUAD, and lung squamous cell carcinoma (LUSC). [2] Although genetic molecular analysis is becoming more common to help clinicians select appropriate target therapies for NSCLC patients, such as EGFR-tyrosine kinase inhibitors (EGFR-TKIs) for EGFR mutant patients, [2] intratumor heterogeneity (ITH) can still lead to therapeutic failure, drug resistance, thus leading to unfavourable prognosis. [3] A recent study found widespread ITH for both somatic mutations and copy-number alterations in early-stage NSCLC patients, which may give useful information for evolutionary tumorigenesis. [4] However, we still lack of the ITH comparison among different subtypes of NSCLC. Moreover, circulating tumor DNA (ctDNA) released by tumor cell into the blood, can originate from any subclonal population within the tumor and therefore has great potential for presenting ITH. [5] Whether ctDNA profile could represent these ITH is still an open question.

**Fig. 1:**
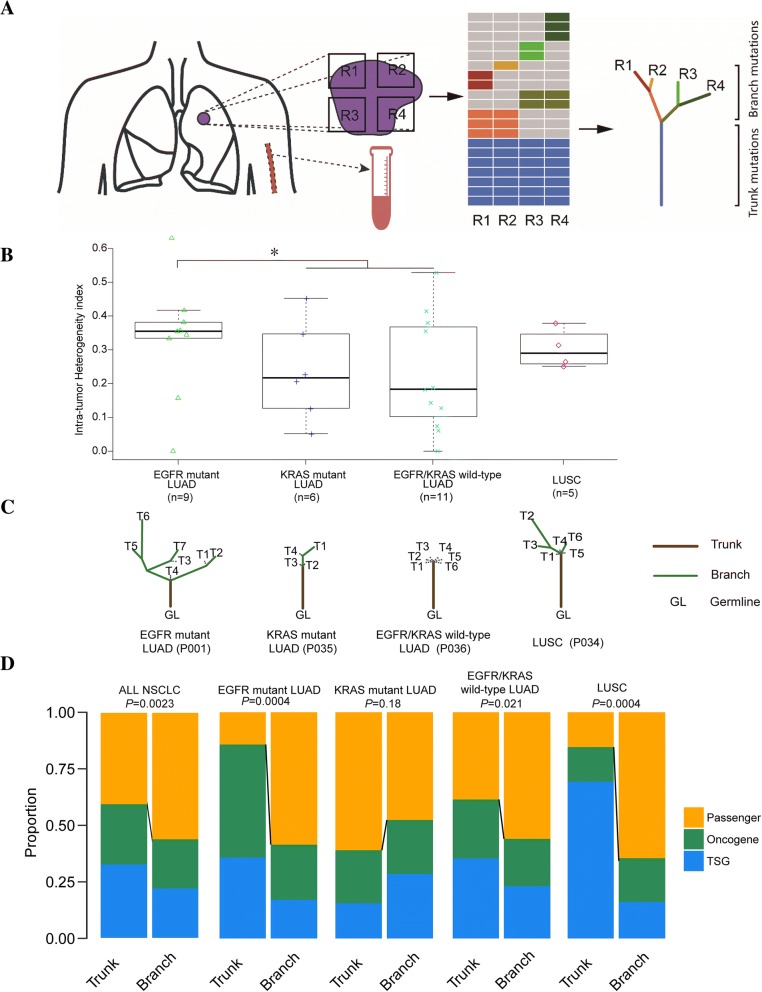
Intratumor heterogeneity analysis by multi-region tissue sequencing. **a**, Overview of the study methodology. **b**, The intratumor heterogeneity comparison among different NSCLC subtypes. **c**, Representative phylogenetic trees of different NSCLC subtypes. The phylogenetic tree was constructed with all somatic mutations. Lengths of trunks and branches were proportional to the numbers of detected mutations. **d**, The proportions of putative driver mutations (oncogenes and tumor suppressor genes) versus passenger mutations on the trunks and branches.

To address these issues, we performed targeted capture sequencing (1021-gene panel, Additional file 1: Table S1) of 181 multi-region tumor tissues and matched ctDNA (Fig. 1a) from 32 operative NSCLC patients **(**Additional file 2: Table S2), including 26 LUAD (9 EGFR-mutant LUAD, 6 KRAS-mutant LUAD and 11 EGFR&KRAS-wild-type LUAD), 5 LUSC and 1 had lymphoepithelioma-like carcinoma (LELC), to compare ITH among different NSCLC subtypes and examine potential value of ctDNA for ITH analysis. ITH is evaluated by ITH index (ITHi). If the somatic genetic alteration is shared by all the tissue regions, it is defined as trunk mutation. Otherwise, it is called branch mutation (Fig. 1a). The ITHi is higher, if the tumor has less trunk mutations. We also constructed phylogenetic trees based on somatic mutations detected in multiple regions (Fig. 1a). Additionally, the cancer genome atlas (TCGA) datasets were downloaded to confirm a high correlation of mutation number between whole exome sequencing (WES) and our panel sequencing (Additional file 3: Figure S1). Besides, WES was performed on 4 randomly selected patients (21 regions) to evaluate the consistency between panel sequencing and WES for ITH and phylogenetic trees (Additional file 4: Figure S2) analysis. Much more details about methods were shown in Additional file 5.

## Results/discussion

### ITH analysis by multi-region tissue sequencing

As shown in the genetic landscape, we identified 437 somatic variations, including 421 SNVs and Indels, 13 CNVs, and 3 gene fusions in overall enrolled patients (Additional file 6: Figure S3). A total of 23 genes were mutated in more than 10% of patients. Mutational prevalence was largely consistent between this study cohort and other two reports of the TCGA and MSK [6] cohorts (Additional file 7: Figure S4). EGFR-mutant LUAD showed significantly higher ITHi than KRAS-mutant LUAD/EGFR&KRAS-wild-type LUAD (*P* = 0.03, Fig. 1b). However, there was no significant difference of ITHi between LUAD and LUSC. Representative phylogenetic trees of different NSCLC subtypes were displayed in Fig. 1c. EGFR-mutant LUAD had the lowest proportion of trunk mutations and the highest proportion of branch mutations, compared to KRAS-mutant LUAD and other NSCLC subtypes, in accordance with the implication of ITHi analysis. Additional file 8: Figure S5 showed the phylogenetic tree for each patient. Generally, in trunk, driver mutations were identified at a higher proportion than passenger mutations (60% vs. 40%, *P* = 0.0023), especially in EGFR-mutant LUAD (86% vs. 14%, *P* = 0.0004) and LUSC (85% vs. 15%, *P* = 0.0004), while this result was opposite in KRAS-mutant LUAD (40% vs. 60%, *P* = 0.18). In branch, the proportions of driver mutations and passenger mutations were similar for each group. Additionally, for driver mutations, the proportions of mutations in oncogenes and tumor suppressor genes (TSG) seemed to be similar both in the trunk (58 and 42%) and branch (49 and 51%) in general. However, oncogene mutations showed higher proportion in EGFR-mutant LUAD compared with TSG, while TSG alterations had a strong enrichment in LUSC compared with oncogene in trunk (Fig. 1d). Besides, driver dominance analysis showed a higher dominance score for EGFR than KRAS and suggested that EGFR mutations tend to have less co-drivers (Additional file 9: Figure S6).

### Tumor-derived mutations by ctDNA sequencing

**Fig. 2:**
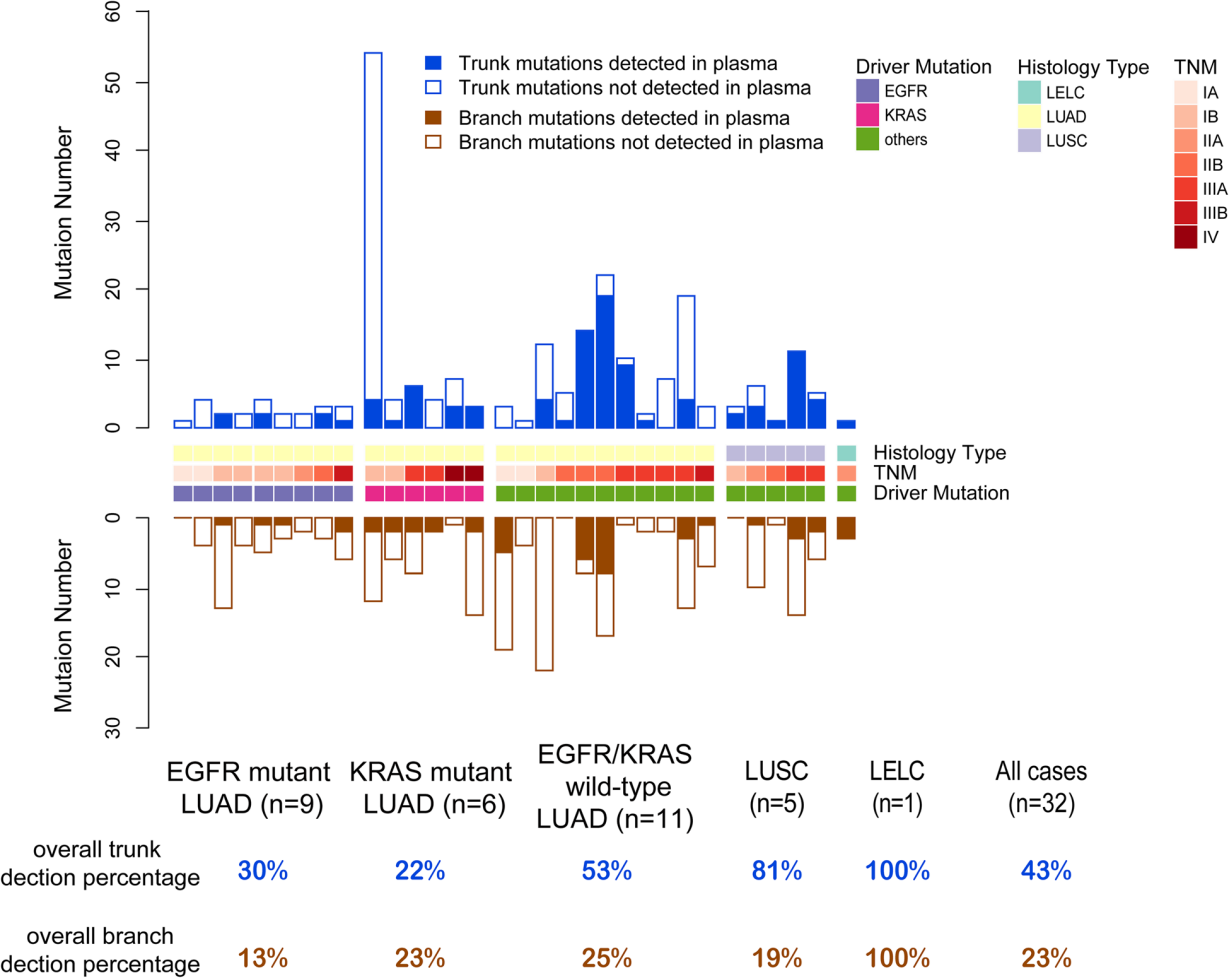
Tumor-derived mutations by ctDNA sequencing.The designations between the two bar charts showed histology type, typical mutant driver genes, and TNM stage of each patient. The table at the bottom of panel describes the overall trunk or branch mutation detection percentage in different subtypes of NSCLC

We also analyzed the distribution of tumor-derived trunk and branch mutations in ctDNA for evaluating its potential application in ITH analysis. A total of 146 tumor-derived mutations were detected with at least one high-quality mutant read in 29 (91%) patients. In general, it was much easier to detect trunk mutations than branch mutations in ctDNA (43% vs. 23%, *P* = 4.53e-6; Fig. 2). As for different NSCLC molecular subtypes, LUSC and EGFR&KRAS-wild-type LUAD had higher proportions for tumor-derived trunk mutations (81 and 53%) than those in EGFR-mutant LUAD (30%) and KRAS-mutant LUAD (22%), while the detections for tumor-derived branch mutations in ctDNA were extremely poor (from 13 to 25%) among all above NSCLC subtypes.

### Translational relevance

EGFR-mutant LUAD showed the highest ITH than KRAS-mutant LUAD, EGFR&KRAS-wild-type LUAD and LUSC, namely the somatic genetic alterations of EGFR-mutant LUAD had the least proportion in trunk (lowest clonal mutation load) and the highest proportion in branch (highest subclonal mutation load). It is known that neoantigens arise as a consequence of tumor-specific mutations. [7] And thus possibly, it might lead to produce less clonal neoantigens and more subclonal neoantigens in EGFR-mutant LUAD. Previous study reported that only clonal neoantigens, instead of subclonal neoantigens, could elicit T cell immunoreactivity and sensitivity to ICIs. [8] Another study has described poorer efficacy for ICI treatment in EGFR-mutant NSCLC compared with EGFR-wild NSCLC. [9] It gave important assumption that the poor efficacy of ICIs in EGFR-mutant LUAD might be due to less clonal neoantigens, which should be explored in the future.

Moreover, the driver dominance score in EGFR-mutant LUAD was higher than that in KRAS-mutant LUAD, which meant that EGFR tended to have less co-drivers than KRAS. These finding indicates blockade of the EGFR-driven pathway may therefore lead to destruction of EGFR-positive cancer, perhaps providing an explanation for the dramatic therapeutic effect of EGFR-TKI for EGFR-mutant LUAD. [2] However, KRAS-mutant LUAD was more complicated owning lower ITH, higher proportions of trunk passenger mutations and less driver dominance score. These results indicate that KRAS maybe not a dominant driver gene. Passenger mutations may also play important roles and collaborate synergistically with driver mutations to trigger tumorigenesis in KRAS-mutant LUAD. Currently, targeted therapy in KRAS-mutant LUAD yields a poor response, [10] which may be due to focusing much more on the driver gene ‘KRAS’ and neglecting of passenger mutations’ effect.

ctDNA analysis showed only LUSC had acceptable detection of tumor-derived trunk mutations, while EGFR&KRAS-wild-type LUAD, EGFR-mutant LUAD and KRAS-mutant LUAD showed unsatisfactory detections of trunk mutations. And the detections for tumor-derived branch mutations in ctDNA were extremely poor among all NSCLC subtypes. As a result, ctDNA profile maybe not an appropriate method to reflect ITH compared to multi-region tumor tissue. Whether circulating tumor cells (CTC) sequencing could have its value for ITH analysis should be explored in the future.

Although the present study showed several interesting findings, our conclusions may be affected by several limitations. First, the low number of patients was a limitation. Our results will be more reliable if we enrolled more patients in this study. Besides, we used panel target capture sequencing (Additional file 10) instead of exome or genome sequencing for analyses, which may have resulted in some degree of missing data. However, we used a pan-cancer panel that included 1021 genes related to tumorigenesis for sequencing analyses, and this panel showed a significant positive correlation with WES in terms of mutation number and ITHi.

## Conclusions

EGFR-mutant LUAD has the highest ITH than other NSCLC subtypes, offering further understanding of tumorigenesis mechanisms among different NSCLC subtypes. Besides, ctDNA maybe not an appropriate method to reflect ITH.

## Additional files

Additional file 1: **Table S1.** List of target regions of the pan-cancer 1021-gene panel. (DOCX 37 kb)

Additional file 2: **Table S2.** Clinical characteristics of the enrolled non-small cell lung cancer patients. (DOCX 14 kb)

Additional file 3: **Figure S1.** The correlations of mutation numbers between panel sequencing and WES in three cohorts. (A) TCGA-LUAD, (B) TCGA-LUSC, (C) Geneplus. Abbreviations: *TCGA* The Cancer Genome Atlas, *LUAD* lung adenocarcinoma, *LUSC* lung squamous cell carcinoma, *WES* wholeexome sequencing. (PDF 124 kb)

Additional file 4: **Figure S2.** The consistency of mutation numbers, ITH and phylogenetic trees between panel sequencing and WES in 4 randomly selected patients. (A) mutation numbers, (B) ITH, (C) phylogenetic trees. Abbreviations: *ITH* intratumor heterogeneity, *WES* wholeexome sequencing. (PDF 259 kb)

Additional file 5: Supplementary Methods. (DOCX 21 kb)

Additional file 6: **Figure S3.** The landscape of genomic alterations in enrolled non-small cell lung cancer patients. Integrated genomic data for 181 regions from 32 NSCLC patients. Overall number of mutations, as well as pathological type and typical mutant driver for each sample, were shown at the top. The group at the top and the patient No. at the bottom indicate contiguous regions from each patient. The percentage of NSCLC regions with an alteration was shown on the left in the order of the mutated genes as listed to the right of the panel. (PDF 276 kb)

Additional file 7: **Figure S4.** The comparison of commonly mutated genes in non-small cell lung cancer among three different cohorts. (PDF 59 kb)

Additional file 8: **Figure S5.** The phylogenetic tree for each patientstratified by different non-small cell lung cancer subtype. Lengths of trunks and branches are proportional to the numbers of mutations acquired. Trunks and branches are shown in different colors. Genes with recurrent putative driver mutations are indicated beside the corresponding branches or trunks. The total number of mutations (n), patient No., and evolution pattern (linear [circle] or branch evolution [star]) are indicated below each tree. TNM stage of each patient is indicated by the colored track above each row of trees. (PDF 389 kb)

Additional file 9: **Figure S6.** Driver dominance score. Driver dominance score measures driver self-sufficiency for each driver gene calculated across 32 patients. It is plotted against the fraction of patients carrying the mutated driver. (PDF 179 kb)

Additional file 10: Supplementary Sequencing datasets. (XLSX 416 kb)

## Abbreviations

ctDNA: circulating tumor DNA
EGFR: Epidermal growth factor receptor
ITH: Intratumor heterogeneity
KRAS: Kirsten rat sarcoma viral oncogene
LUAD: Lung adenocarcinoma
LUSC: Lung squamous cell carcinoma
NSCLC: Non-small-cell lung cancer
TCGA: The Cancer Genome Atlas
WES: Whole exome sequencing;
