# Supplementary material for: Intratumor heterogeneity comparison among different subtypes of non-small-cell lung cancer through multi-region tissue and matched ctDNA sequencing
Source: Mol Cancer. 2019 Jan 9;18:7. doi: 10.1186/s12943-019-0939-9 (PMC6325778; doi:10.1186/s12943-019-0939-9)
Supplement: Supplementary file 2 — Table S2. Clinical characteristics of the enrolled non-small cell lung cancer patients. (DOCX 14 kb) [file 12943_2019_939_MOESM2_ESM.docx]

**Table S2. Clinical characteristics of the enrolled NSCLC patients.**

| **Characteristic** | | **Patients(n)** |
| --- | --- | --- |
| Age median(range): 57(45-79) | <60 | 18 |
|  | ≥60 | 14 |
| Gender | Male | 25 |
|  | Female | 7 |
| Histology | Adenocarcinoma | 26 |
|  | Squamous cell carcinoma | 5 |
|  | Lymphoepithelioma-like carcinoma | 1 |
| TNM stage^*^ | Ia | 4 |
|  | Ib | 8 |
|  | IIa | 3 |
|  | IIb | 5 |
|  | IIIa | 8 |
|  | IIIb | 2 |
|  | IV^#^ | 2 |
| Lymph node metastasis | Yes | 15 |
|  | No | 17 |
| Drinking status | drinker | 16 |
|  | non-drinker | 16 |
| Smoking status | smoker | 21 |
|  | non-smoker | 11 |
| Neoadjuvant therapy | Yes^$^ | 2 |
|  | No | 30 |

* AJCC Version 7

# Both patients had solitary brain metastasis and received brain radical surgery treatment.

$ Pemetrexed plus cisplatin, 2 cycles
