# Supplementary material for: Intratumor heterogeneity comparison among different subtypes of non-small-cell lung cancer through multi-region tissue and matched ctDNA sequencing
Source: Mol Cancer. 2019 Jan 9;18:7. doi: 10.1186/s12943-019-0939-9 (PMC6325778; doi:10.1186/s12943-019-0939-9)
Supplement: Supplementary file 3 — Figure S1. The correlations of mutation numbers between panel sequencing and WES in three cohorts. (A) TCGA-LUAD, (B) TCGA-LUSC, (C) Geneplus. Abbreviations: TCGA The Cancer Genome Atlas, LUAD lung adenocarcinoma, LUSC lung squamous cell carcinoma, WES wholeexome sequencing. (PDF 124 kb) [file 12943_2019_939_MOESM3_ESM.pdf]

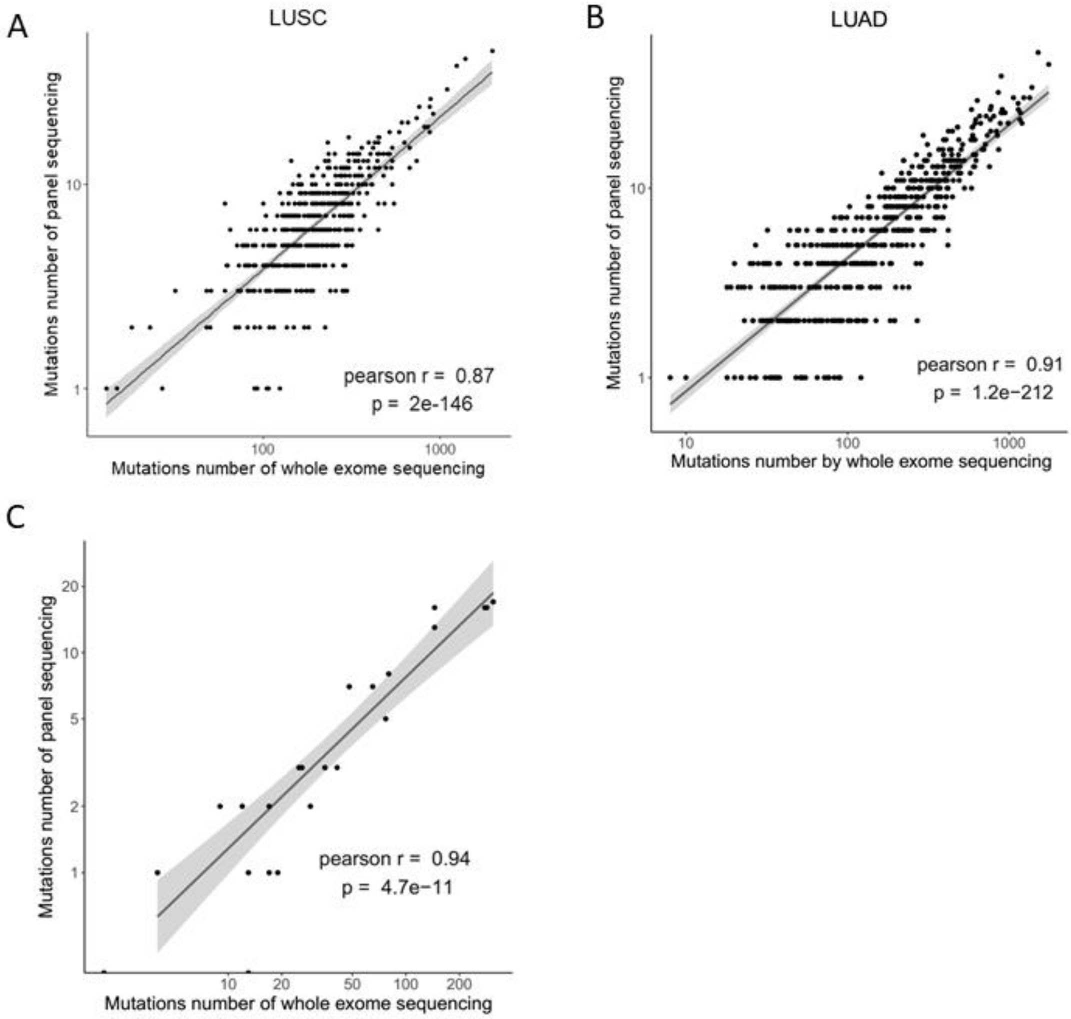

FigureS1. The correlations of mutation numbers between panel sequencing andWES in three cohorts.

(A)TCGA-LUAD, **(B)**TCGA-LUSC, **(C)**Geneplus.

Abbreviations: TCGA, The Cancer Genome Atlas; LUAD, lung adenocarcinoma; LUSC, lung squamous cell carcinoma; WES, wholeexome sequencing
