# Supplementary material for: Intratumor heterogeneity comparison among different subtypes of non-small-cell lung cancer through multi-region tissue and matched ctDNA sequencing
Source: Mol Cancer. 2019 Jan 9;18:7. doi: 10.1186/s12943-019-0939-9 (PMC6325778; doi:10.1186/s12943-019-0939-9)
Supplement: Supplementary file 4 — Figure S2. The consistency of mutation numbers, ITH and phylogenetic trees between panel sequencing and WES in 4 randomly selected patients. (A) mutation numbers, (B) ITH, (C) phylogenetic trees. Abbreviations: ITH intratumor heterogeneity, WES wholeexome sequencing. (PDF 259 kb) [file 12943_2019_939_MOESM4_ESM.pdf]

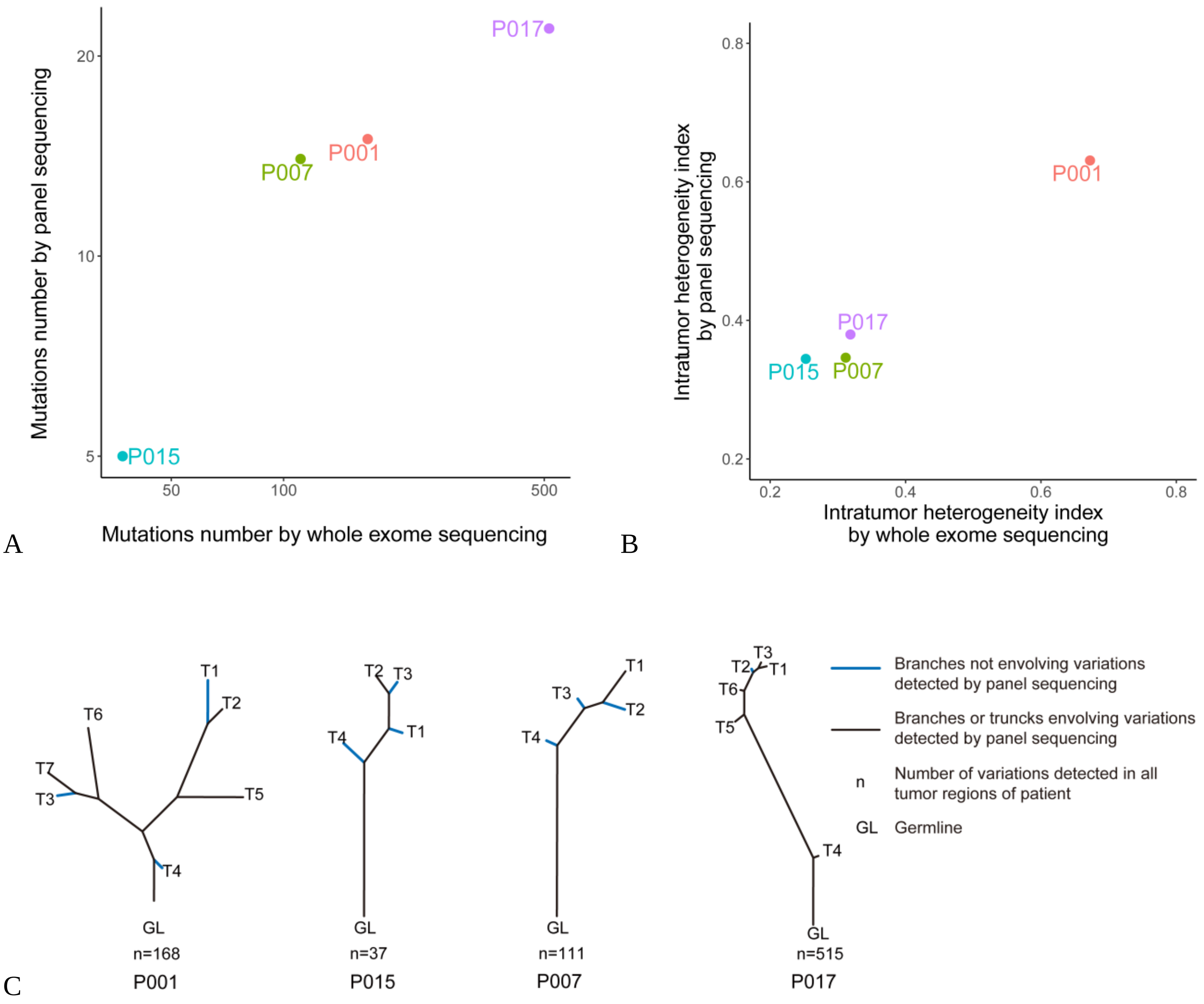

Figure S2. The consistency of mutation numbers, ITH and phylogenetic trees between panel sequencing and WES in 4 randomly selected patients.

(A) mutation numbers, **(B)** ITH, **(C)** phylogenetic trees

Abbreviations: ITH, intratumor heterogeneity; WES, whole exome sequencing
