# Supplementary material for: Intratumor heterogeneity comparison among different subtypes of non-small-cell lung cancer through multi-region tissue and matched ctDNA sequencing
Source: Mol Cancer. 2019 Jan 9;18:7. doi: 10.1186/s12943-019-0939-9 (PMC6325778; doi:10.1186/s12943-019-0939-9)
Supplement: Supplementary file 5 — Supplementary Methods. (DOCX 21 kb) [file 12943_2019_939_MOESM5_ESM.docx]

**Supplementary Methods**

**Study populations and ethics approval**

All enrolled patients received pulmonary surgery at the Sun Yat-Sen University Cancer Center from June 2016 to March 2017. We collected multi-region tumor tissues with matched peripheral blood and clinical information from each patient. All patients provided written informed consent. This study was approved by the Institutional Review Board (IRB) of Sun Yat-Sen University Cancer Center (IRB number B2017-067-01).

**Targeted capture sequencing**

Peripheral blood was collected in EDTA Vacutainer tubes (BD Diagnostics, Franklin Lakes, NJ, USA) and processed within 3 h. Plasma was separated by centrifugation at 2,500 ×g for 10 min, transferred to microcentrifuge tubes, and then centrifuged at 16,000×g for 10 min to remove remaining cell debris. Peripheral blood lymphocytes (PBLs) from the first centrifugation step were used for the extraction of germline genomic DNA (gDNA). The gDNA of PBL and tissue samples were extracted using the DNeasy Blood & Tissue Kit (Qiagen, Hilden, Germany).To detect ctDNA, circulating cell-free DNA (cfDNA) was isolated from 0.6–1.8 mL plasma using QIAamp Circulating Nucleic Acid Kit (Qiagen). DNA concentration was measured using a Qubit fluorometer (Invitrogen, Carlsbad, CA, USA) and the Qubit dsDNA HS (High Sensitivity) Assay Kit (Invitrogen). The size distribution of the cfDNA was assessed using an Agilent 2100 BioAnalyzer and the DNA HS kit (Agilent Technologies, Santa Clara, CA, USA). All DNA extractions were performed according to the manufacturer’s instructions. Sequencing libraries of both cfDNA and gDNA were constructed with the KAPA DNA Library Preparation Kit (Kapa Biosystems, Wilmington, MA, USA) according to the manufacturer’s protocol. DNA sequencing was performed using the HiSeq 3000 Sequencing System (Illumina, San Diego, CA, USA) with 2×101-bp paired-end reads.

Targeted capture sequencing revealed a mean effective depth of coverage of 816 ×in multi-region tissue samples and 1901 × in ctDNA samples. Single nucleotide variants (SNVs) were called using MuTect(version 1.1.4) and NChot, a software developed in-house to review hotspot variants. Small insertions and deletions (Indels) were called by GATK. Somatic copy-number alterations were identified with CONTRA (v2.0.8). Copy number variations (CNV) was expressed as the ratio of adjusted depth between ct DNA and germline DNA. Mutations were considered a candidate somatic mutation only when (i) the mutation was detected in at least 5 high-quality reads containing the particular base,(ii) the mutation was not present in >1% of the population in the 1000 Genomes Project (version phase 3) or dbSNP databases (The Single Nucleotide Polymorphism Database, version dbSNP 137), and (iii) the mutation was not present in a local database of normal samples. High-quality reads were selected with Phred score ≥30, mapping quality≥30, and a lack of paired-end reads bias. For tumor somatic mutations, the mutant allele must be present in ≥1% of reads. The candidate variants were all manually verified in the Integrative Genomics Viewer. Driver gene list was downloaded from IntoGene (https://www.intogen.org/). The oncogene and tumor suppressor genes (TSG) were classified based on COSMIC database (https://cancer.sanger.ac.uk/cosmic).

**WES analysis**

DNA libraries were hybridized to SeqCap EZ Exome 64M (Roche NimbleGen, Madison, WI, USA) according to the manufacturer's instructions. Sequencing was carried out with the HiSeq 3000 Sequencing System (Illumina, San Diego, CA, USA) with 2×150-bp paired-end reads. The terminal adaptor sequences and low-quality reads were removed from the raw data. BWA (version 0.7.12-r1039) was employed to align the clean reads to the reference human genome (hg19). Picard (version 1.98) was used to mark PCR duplicates. Realignment and recalibration was performed using GATK (version 3.4-46-gbc02625). Amean effective depth of coverage of 139× was obtained.

**ITH evaluation**

To eliminate bias introduced by the number of tumor regions, ITH index (ITHi) was evaluated for each patient based on the presence of each detected genetic variation (SNV, indel, gene fusion, and CNV) in tumor regions with more than one variation and expressed as the mean Jaccard distance between variation sets of each of two regions. Patients were excluded from ITHi analysis when (i) only one region contained more than one mutation or (ii) only one mutation was detected in all regions.

$$\begin{aligned} ITH index=\frac{1}{C_{\left| R \right|}^{2}}\sum_{1\leq i<j\leq\left| R \right|} d_{J}\left( V_{i},V_{j} \right)\#\left（ 1 \right） \end{aligned}$$

$$\begin{aligned} d_{J}\left( V_{i},V_{j} \right)=1-J\left( V_{i},V_{j} \right)=1-\frac{\left| V_{i}\cap V_{j} \right|}{\left| V_{i}\cup V_{j} \right|}\#\left（ 2 \right） \end{aligned}$$

where $R$ is the set of regions of one patients in which at least one variation was detected, and $|R|$ is the number of regions in $R$. $V_{i}$ and $V_{j}$indicate the set of variations detected in the $i$st and $j$st region in $R$, respectively. $d_{J}\left( V_{i},V_{j} \right)$ is the Jaccard distance between $V_{i}$ and $V_{j}$, and this distance measures dissimilarity between $V_{i}$ and $V_{j}$ and is complementary to the Jaccard coefficient ($J\left( V_{i},V_{j} \right)$) and obtained by subtracting the Jaccard coefficient from 1. ITHis range from 0 (lowest ITH) to 1 (highest ITH). If the tumor has less shared somatic genetic alterations (SNV, indel, gene fusion, CNV) after multi-region sequencing, namely less trunk mutations, the ITHi of this tumor will be higher. Otherwise, if the tumor has more trunk mutations, the ITHi of this tumor will be lower.

**Driver dominance score**

Driver dominance score was calculated for all known driver gene in all 32 NSCLC patients as reported previously (Nahar R, Zhai W, Zhang T, et al. Elucidating the genomic architecture of asian egfr-mutant lung adenocarcinoma through multi-region exome sequencing. Nature communications 2018;9:216). It implies the self-sufficiency of driver genes. Mutations which possess higher capability to drive tumorigenesis (namely higher score) would has fewer co-occurring driver mutations.

**Phylogenetic tree analysis**

Non-synonymous mutations annotated by ANNOVAR were used in phylogenetic tree construction. Based on SNV, indel, gene fusion, and CNV that were identified in each tumor region of the same patient, a phylogenetic tree was constructed using the R package “phangorn” with the methods “pratchet” and “acctran”. Trees were built using binary presence/absence matrices built from the regional distribution of variants within the tumor. Mutations can be considered ‘trunk’ if it occurred in all regions of the tumor. The length of each branch of the trees was calculated according to the number of mutations on the branch.

**Statistical analysis**

Pearson correlation analysis was used to test the linear association between WES and panel sequencing for mutation number. The Student’s *t*-test was employed to compare ITHi in different groups. The Fisher’s exact test was performed to analyze the difference between driver and passenger mutations in trunk or branch. All statistical analyses were performed with SPSS (v.21.0; STATA, College Station, TX, USA) or GraphPad Prism (v. 6.0; GraphPad Software, La Jolla, CA, USA) software. Statistical significance was defined as a two-sided *P*<0.05.
