# Supplementary material for: Intratumor heterogeneity comparison among different subtypes of non-small-cell lung cancer through multi-region tissue and matched ctDNA sequencing
Source: Mol Cancer. 2019 Jan 9;18:7. doi: 10.1186/s12943-019-0939-9 (PMC6325778; doi:10.1186/s12943-019-0939-9)
Supplement: Supplementary file 6 — Figure S3. The landscape of genomic alterations in enrolled non-small cell lung cancer patients. Integrated genomic data for 181 regions from 32 NSCLC patients. Overall number of mutations, as well as pathological type and typical mutant driver for each sample, were shown at the top. The group at the top and the patient No. at the bottom indicate contiguous regions from each patient. The percentage of NSCLC regions with an alteration was shown on the left in the order of the mutated genes as listed to the right of the panel. (PDF 276 kb) [file 12943_2019_939_MOESM6_ESM.pdf]

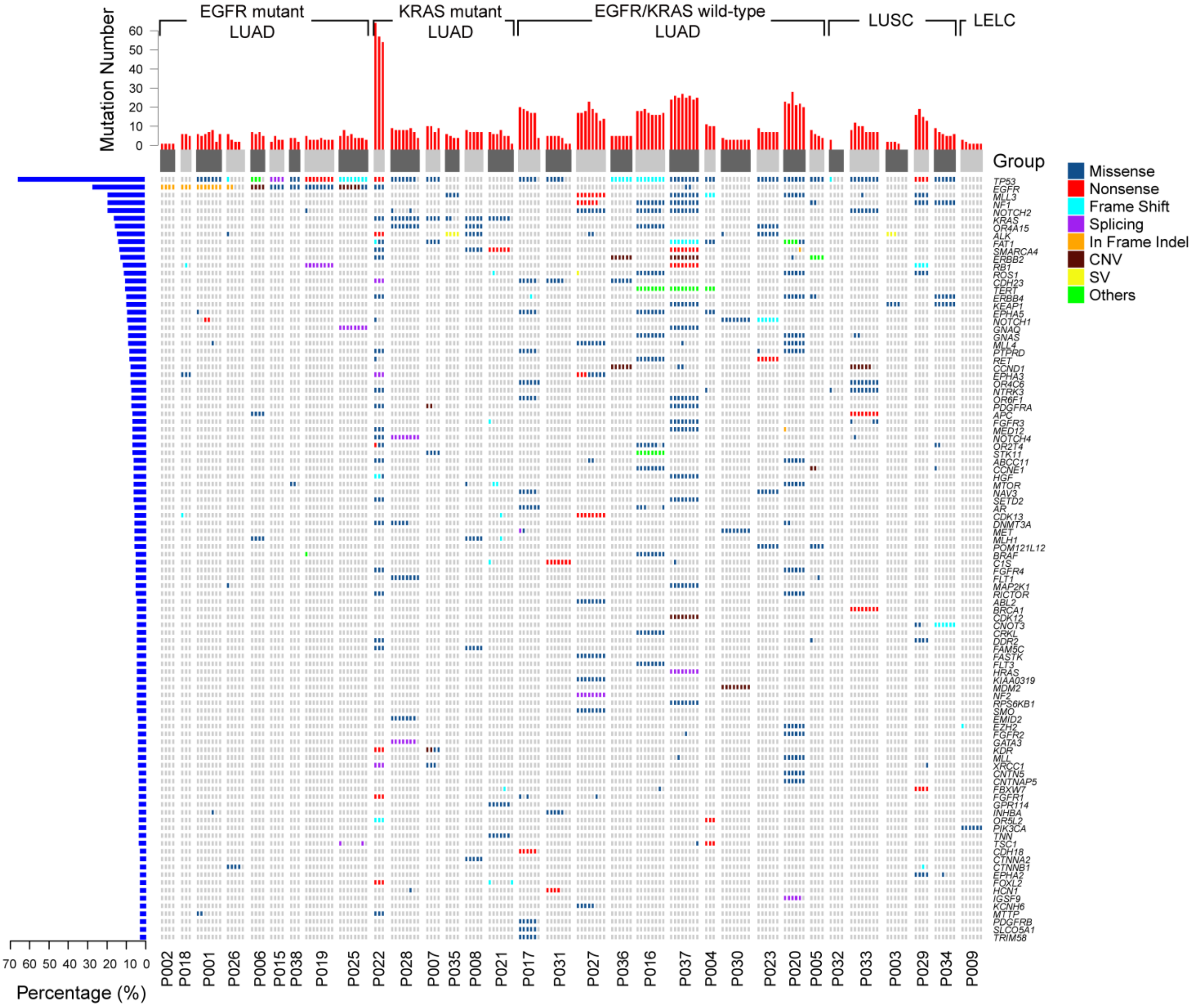

Figure S3. The landscape of genomic alterations in enrolled NSCLC patients.

Integrated genomic data for 181 regions from 32 NSCLC patients. Overall number of mutations, as well as pathological type and typical mutant driver for each sample, were shown at the top. The group at the top and the patient No. at the bottom indicate contiguous regions from each patient. The percentage of NSCLC regions with an alteration was shown on the left in the order of the mutated genes as listed to the right of the panel.

Abbreviations: NSCLC: non-small-cell lung cancer
