# Supplementary material for: Intratumor heterogeneity comparison among different subtypes of non-small-cell lung cancer through multi-region tissue and matched ctDNA sequencing
Source: Mol Cancer. 2019 Jan 9;18:7. doi: 10.1186/s12943-019-0939-9 (PMC6325778; doi:10.1186/s12943-019-0939-9)
Supplement: Supplementary file 7 — Figure S4. The comparison of commonly mutated genes in non-small cell lung cancer among three different cohorts. (PDF 59 kb) [file 12943_2019_939_MOESM7_ESM.pdf]

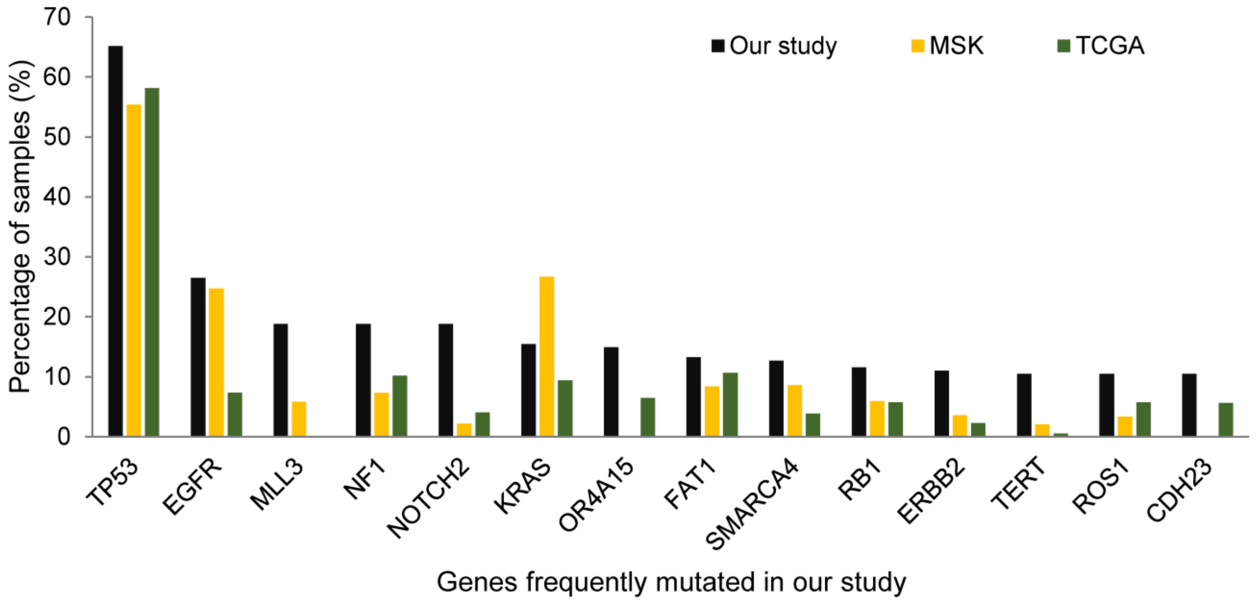

Figure S4. The comparison of commonly mutated genes in NSCLC among three different cohorts.

Abbreviations: NSCLC: non-small-cell lung cancer
