# Supplementary material for: Intratumor heterogeneity comparison among different subtypes of non-small-cell lung cancer through multi-region tissue and matched ctDNA sequencing
Source: Mol Cancer. 2019 Jan 9;18:7. doi: 10.1186/s12943-019-0939-9 (PMC6325778; doi:10.1186/s12943-019-0939-9)
Supplement: Supplementary file 8 — Figure S5. The phylogenetic tree for each patientstratified by different non-small cell lung cancer subtype. Lengths of trunks and branches are proportional to the numbers of mutations acquired. Trunks and branches are shown in different colors. Genes with recurrent putative driver mutations are indicated beside the corresponding branches or trunks. The total number of mutations (n), patient No., and evolution pattern (linear [circle] or branch evolution [star]) are indicated below each tree. TNM stage of each patient is indicated by the colored track above each row of trees. (PDF 389 kb) [file 12943_2019_939_MOESM8_ESM.pdf]

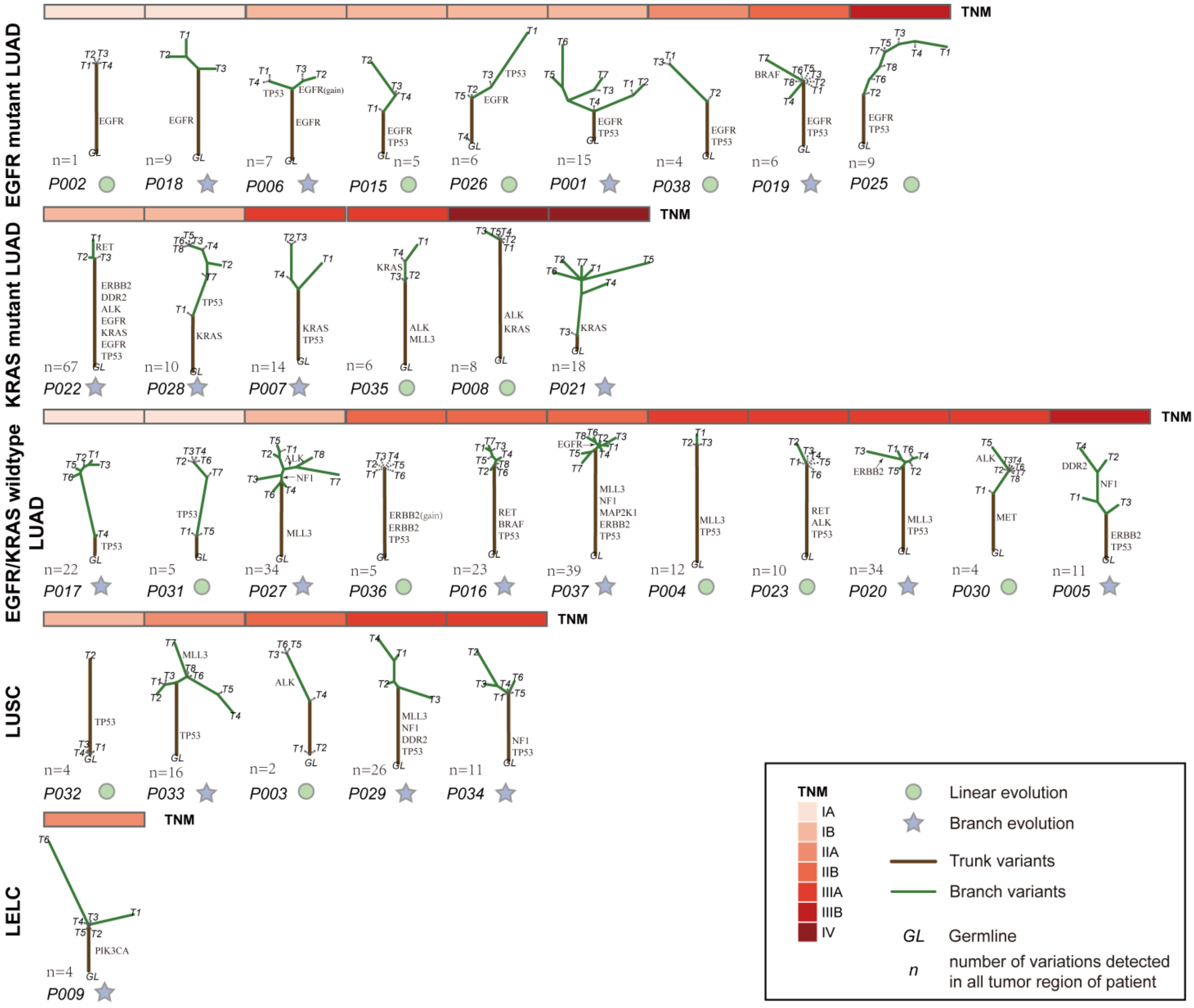

Figure S5. The phylogenetic tree for each patient stratified by different NSCLC subtype.

Lengths of trunks and branches are proportional to the numbers of mutations acquired. Trunks and branches are shown in different colors. Genes with recurrent putative driver mutations are indicated beside the corresponding branches or trunks. The total number of mutations (n), patient No., and evolution pattern (linear [circle] or branch evolution [star]) are indicated below each tree. TNM stage of each patient is indicated by the colored track above each row of trees.

Abbreviations: NSCLC: non-small-cell lung cancer
