# Supplementary material for: Intratumor heterogeneity comparison among different subtypes of non-small-cell lung cancer through multi-region tissue and matched ctDNA sequencing
Source: Mol Cancer. 2019 Jan 9;18:7. doi: 10.1186/s12943-019-0939-9 (PMC6325778; doi:10.1186/s12943-019-0939-9)
Supplement: Supplementary file 9 — Figure S6. Driver dominance score. Driver dominance score measures driver self-sufficiency for each driver gene calculated across 32 patients. It is plotted against the fraction of patients carrying the mutated driver. (PDF 179 kb) [file 12943_2019_939_MOESM9_ESM.pdf]

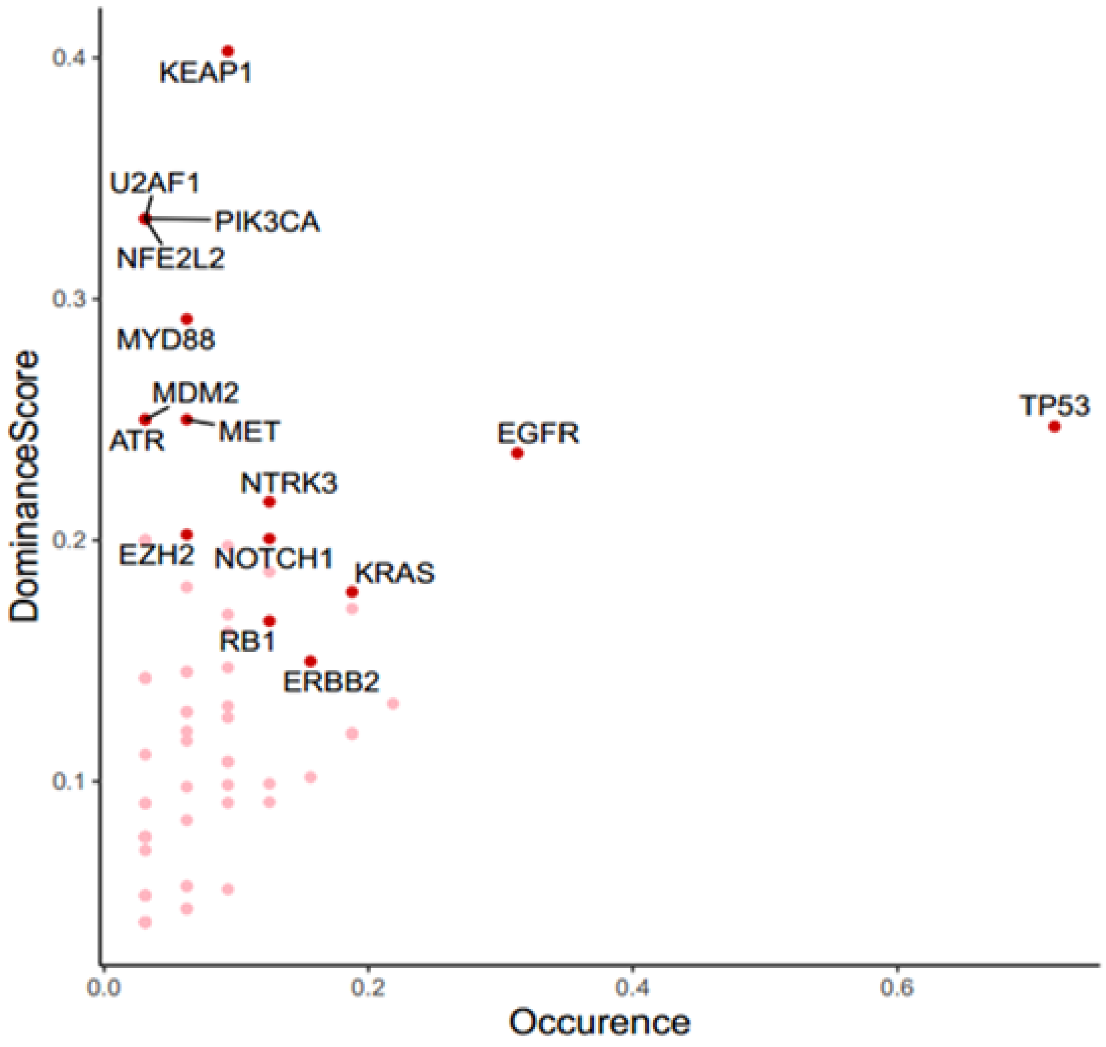

Figure S6. Driver dominance score.

Driver dominance score measures driver self-sufficiency for each driver gene calculated across 32 patients. It is plotted against the fraction of patients carrying the mutated driver.
